# Dose-Related Regulatory Effect of Raspberry Polyphenolic Extract on Cecal Microbiota Activity, Lipid Metabolism and Inflammation in Rats Fed a Diet Rich in Saturated Fats

**DOI:** 10.3390/nu15020354

**Published:** 2023-01-10

**Authors:** Bartosz Fotschki, Ewelina Cholewińska, Katarzyna Ognik, Michał Sójka, Joanna Milala, Joanna Fotschki, Wiesław Wiczkowski, Jerzy Juśkiewicz

**Affiliations:** 1 Division of Food Science, Institute of Animal Reproduction and Food Research, Tuwima 10, 10-748 Olsztyn, Poland; 2 Department of Biochemistry and Toxicology, Faculty of Animal Sciences and Bioeconomy, University of Life Sciences in Lublin, Akademicka 13, 20-950 Lublin, Poland; 3 Institute of Food Technology and Analysis, Łódź University of Technology, Stefanowskiego 4/10, 90-924 Łódź, Poland

**Keywords:** raspberry, polyphenols, large gut, liver, lipid metabolism, ellagic acid, obesity, rats

## Abstract

The amount of berry polyphenols required to exert health-promoting effects seems to be difficult to achieve by fresh fruit ingestion, so polyphenol-rich extracts could be considered a dietary alternative. In the present study, laboratory rats were fed high-fat diets supplemented with 0.1 or 0.3% raspberry polyphenols from pomace, with the former dose reflecting the amount of polyphenols consumed with a glass of fresh raspberries. It was hypothesized that beneficial changes in blood and hepatic tissue related to lipid metabolism would accompany both treatments, but the health-promoting effect would be more noticeable with the higher dose of extract. This hypothesis was confirmed, and the high dose of raspberry polyphenols was better than the low dose extract in terms of decreased epididymal white adipose tissue weight, hepatic triglyceride content, PPARγ and SREBP-1c expression in the liver, and plasma IL-6 concentration, as well as increased acetic acid concentration in the cecal digesta. These effects might be partially associated with the enhanced content of ellagitannin and anthocyanin metabolites found in the blood plasma of rats administered the high dose of the extract. The results showed that this extract could be considered a dietary vehicle to provide an amount of raspberry polyphenols that could promote health.

## 1. Introduction

A diet enriched with fruits reduces the risk of many chronic diseases. One of the most consumed types of fruit in both fresh and processed form is berries, including raspberries. These fruits are characterized by high contents of phenolic compounds, e.g., ellagitannins (ETs), anthocyanins (ACYs) and flavanols [1]. Diets enriched with these compounds might enhance the oxidative status of organisms [2]. In addition to their strong antioxidant properties, polyphenols from raspberries may exert numerous health-promoting effects related to the regulation of microbial activity in the gastrointestinal tract, lipid metabolism and inflammation [2,3,4]. Moreover, these polyphenols may also modulate hepatic signals related to disorders characteristic of obesity [5]. Most berry-related nutritional studies have focused on the health effects of fresh fruits, whereas their juices and polyphenolic extracts contain mostly ACYs [6,7,8]. Another valuable source of biologically active compounds is raspberry pomaces. When these fruits are processed, particularly to produce concentrated fruit juices, a significant portion of the bioactive compounds remains in the pomace, the nutritional and health-promoting properties of which are not yet sufficiently known. This byproduct is a rich source of polyphenols, mainly ETs, flavanols and, in a small amount, ACYs [9]. However, increased supplementation with polyphenols, particularly tannins (including ETs), might also negatively affect the bioavailability of vitamins and minerals and inhibit the activity of digestive enzymes as well as bacterial enzymes in the gastrointestinal tract [10,11]. Therefore, determining the ideal level of supplementation with polyphenolic extracts containing ETs seems to be paramount to optimally utilize their health-promoting properties.

The favorable effect of dietary polyphenols is associated with microbiota activity in the gastrointestinal tract and their ability to metabolize polyphenols into compounds with greater bioavailability and activity in comparison to their native forms [12,13]. Among all polyphenols found in raspberry pomace, ETs are the most common [9,13]. ETs are very complex molecules that cannot be absorbed by the gastrointestinal tract without undergoing hydrolysis by intestinal and microbial enzymes. Therefore, most of these bioactive compounds reach the large intestine, where as a result of bacterial enzymatic activity, they undergo a metabolic transformation leading to the formation of ellagic acid and various forms of urolithins [14,15]. In addition to the generation of urolithins in almost all mammals after ETs are consumed, there are also some animal species (e.g., pigs and rats) that are able to produce dehydroxyellagic acid derivatives, namely, nasutins [16]. After absorption by the intestine into the bloodstream, raspberry polyphenolic metabolites are first transported to the liver, where they can interact with molecular mechanisms involved in the regulation of metabolism, inflammation and oxidative stress [4,17]. One of the mechanisms that might be activated by raspberry polyphenols is related to the regulation of peroxisome proliferator-activated receptors (PPARs) and sterol regulatory element-binding protein 1c (SREBP-1c) in the liver [18,19]. These molecular factors are responsible for modulating energy metabolism, inflammation, fatty acid oxidation, fat storage and lipogenesis in the liver [20].

The health-promoting effects associated with the consumption of berry polyphenols are unquestioned among researchers, but the amount of daily ingested raspberry polyphenolic extract from pomace that can effectively produce these benefits has not yet been fully examined. In the present experiment, rats fed a high-fat diet for 30 days were subjected to two different doses of dietary raspberry pomace extract, resulting in 0.1% and 0.3% polyphenolic content in their diets. The former treatment (0.1%) reflects the polyphenolic contents available after consumption of a single portion (one glass, approximately 100–120 g) of fresh fruit. It was hypothesized that both raspberry pomace extract treatments would cause beneficial changes in lipid metabolism in the blood and hepatic tissue, and those effects would be more pronounced after treatment with the high dose of extract. If the proposed hypothesis was true, it would justify the production of berry extract on a large scale to deliver an efficacious product (i.e., a workable way to consume a dose of polyphenols that has proven health effects in an organism) that could be available to a wide consumer base.

## 2. Materials and Methods

### 2.1. Raspberry Extract Preparation

The waste product (fruit pomace) following the production of juice from the raspberry variety Polana was the source of the polyphenolic extract used in the feeding experiment conducted on laboratory rats (details below). To maintain the bioactivity of the molecules of the product, the pomace was frozen and kept at 18 °C until extract preparation. The details of the pomace production process were provided by Fotschki et al. (2018) [5]. To obtain a raspberry extract rich in polyphenolic constituents, the fruit pomace was extracted three times with the aid of a water-acetone mix (*v*/*v*, 70/30). The exact description of the extract preparation is available in the paper of Fotschki et al. (2021) [21]. Briefly, the obtained water-acetone extraction pulp was filtered through a Hobrafit S40N cellulose filter (Hobra-Školnik S.R.O., Broumov, Czech Republic). The acetone fraction was subsequently removed using a Heidolph Hei-Vap evaporator (Heidolph, Schwabach, Germany) at 60 °C and a pressure of 135 mbar. After repeated filtration through a Hobrafit S40N cellulose filter, the acetone-free fraction was purified in eight cycles on an Amberlite XAD 1600 sorption bed with the aid of different ethanol solutions (details in Fotschki et al., 2021 [21]). The fractions from these eight cycles were combined, and the ethanol was removed with the aid of a Heidolph Hei-Vap Distimatic evaporator (60 °C, 125 mbar). Subsequently, the extract was further concentrated (60 °C, 72 mbar) and slowly freeze-dried (48 h, −36 °C). Finally, a purified red powder was obtained and analyzed with respect to its chemical composition.

### 2.2. Chemical Analyses of the Raspberry Extract

The chemical composition of the preparations was determined by AOAC methods (2005) [22] (Table 1) using the following procedures: dry matter and ash content, 940.26; protein content, 920.152; crude fat, 930.09; and total dietary fiber, 985.29. The contents of sucrose, fructose, glucose, and other saccharides were determined after desalination of the raspberry extract solution in water (5 mg/mL) anion-exchange column chromatography (two parts Amberlite IRA 400 anion exchanger purchased from Sigma Aldrich (Germany), one part Amberlite IR 120 cation exchanger also purchased from Sigma Aldrich (Germany)). Analysis was performed using an HPLC system (Knauer, Berlin, Germany) with a refractive index (RI) detector and a BIO-RAD Aminex HPX-87C column (300 × 7.8 mm; Phenomenex, Torrance, CA, USA), with a flow rate of 0.5 mL/min at 85 °C. Sucrose (limit of detection of 0.08 mg/mL), glucose (limit of detection of 0.08 mg/mL), and fructose (limit of detection of 0.04 mg/mL) were used as standards.

### 2.3. Analysis of the Raspberry Extract Polyphenols

The contents and compositions of ETs and ACYs in the raspberry extract were analyzed as previously described [5]. Briefly, to characterize the ET and ACY levels, the extract was mixed with methanol, where in the case of ETs, 40 mg of extract was mixed with 10 mL of methanol, and for ACYs, 75 mg of extract was mixed with the same amount of methanol. A Knauer Smartline HPLC system with a diode array detector (DAD) was used for analysis. The separation of ETs and ACYs was performed with the aid of Gemini C18 columns (Phenomenex, Torrance, CA, USA) for ETs (250 × 4.6 mm; 5 μm) and ACYs (150 × 4.6 mm; 5 μm). The ET content was analyzed at 250 nm using the following standards: ellagic acid (Extrasynthese, Genay, France), lambertianin C and sanguiin H-6, where the latter two were obtained as described by Fotschki et al. (2021) [21]. ACY detection was performed at 520 nm and cyanidin-3-O-glucoside (Extrasynthese, Genay, France) was used as the standard. The identification conditions were described by Fotschki et al. (2018) [5] and performed with a liquid chromatography–mass spectrometry (LC–MS) instrument (Q Exactive Orbitrap, Thermo Fisher Scientific).

The proanthocyanidin content in the extract was analyzed via acid-catalyzed degradation of the polymeric molecules in excess phloroglucinol as described in detail by Sójka et al. (2013) [23]. The chromatographic system was provided by Shimadzu Co. (Tokyo, Japan) and consisted of an RF-10Axl detector, LC-20AD pump, DGU-20ASR degasser, CTO-10AS thermostat, and SIL-20AC autosampler. Standard curves of (−)-epicatechin and (+)-catechin were prepared to characterize the terminal units, and the (−)-epicatechin-phloroglucinol adduct was prepared for extender unit characterization. Free (−)-epicatechin and (+)-catechin were identified under the same HPLC conditions described above. The average degree of polymerization (DP) was defined as the molar ratio of all of the flavan-3-ol units to (−)-epicatechin and (+)-catechin.

### 2.4. In Vivo Experiment

This study involved Wistar rats, as they are well-established model animals for nutritional and biochemical studies, including their gastrointestinal response to nutritional interventions and systemic (metabolic) changes observed upon dietary supplementation with bioactive plant components. The feeding study was conducted on 32 adult male outbred Wistar rats (Cmdb:Wi CMDB) of approximately 3 months of age, weighing 382 ± 22.4 g. All procedures and manipulations performed on living animals were performed with respect to the rules described in the 2010/63/European Union Directive and were accepted by the Local Ethics Committee for Animal Experiments in Olsztyn, Poland (application approval no. 51/2016). The rats were randomly subjected to one of four dietary treatments with eight animals in each group. The rats were kept for 30 days in individual cages that met the size requirements for living adult laboratory rats and were fed ad libitum semipurified diets with free access to tap water. The animal laboratory room was windowless with a constant inside temperature of 22 ± 1 °C, controlled relative air humidity of 50–70%, a 12-hour day (light) and 12-hour night (dark) cycle, and 15 air room changes every 60 min. The detailed compositions of the diets used in the study are presented in Table 2.

The animals were properly monitored and observed by highly trained staff and a veterinarian, taking into account their wellbeing and health parameters. The humane endpoints of in vivo studies described in Directive 2010/63/EU were strictly followed in the present experiment. The researcher responsible for the experimental design and the veterinarian were briefed as soon as possible when any signs of distress or pain were observed in an animal. For 30 days, the animals were subjected to the following dietary treatments: the C rats were fed the control low-fat diet containing 2% rapeseed oil and 6% lard; the HF rats were provided a diet enriched with 2% rapeseed oil and 23% lard; and the HF + 0.1PP and HF + 0.3PP groups were subjected to the HF diet for 30 days but supplemented with 0.1 and 0.3% raspberry extract that had been added at the expense of the dietary maize starch preparation. All diets were similar with respect to the casein, DL-methionine, choline chloride, vitamin mixture, mineral mixture, and sucrose contents. The control diet contained 5% cellulose and 0.3% cholesterol, while the three HF diets contained 3% cellulose and 1.0% cholesterol. Maize starch was added up to 100% of each diet. The rats fed the control (C) diet obtained 20 kcal% from dietary protein, 20 kcal% from fat, and 60 kcal% from carbohydrates. The dietary high-fat treatments were denser in energy (by 26.8%), and the dietary fat provided 50 kcal%, while protein provided 15 kcal% and carbohydrates provided 35 kcal%. The diets were composed of well-characterized ingredients and were kept at 4 °C in containers designated for food during the entire experimental feeding stage. The applied dietary treatments were prepared following the rules provided by Reeves (1997) [24] and accepted by the American Institute of Nutrition.

The rats were individually monitored for body weight at the start and the termination of the study, while diet consumption was checked daily. Before termination of the study, the rats were deprived of food for 8 h but had free access to water. On the last day, the animals were subjected to body fat and lean tissue content analyses with the aid of a time-domain nuclear magnetic resonance (NMR) protocol (Minispec LF90II Bruker, Bremen, Germany). The principle of this analysis is that the tissue contrast is high between fat and muscle based on the relative relaxation times. In the applied NMR protocol, animal stress is minimized, as anesthesia is not needed. Moreover, rat stress is further decreased by the use of red restrainers due to rodent dichromatic color perception. After NMR analyses, the rats were anaesthetized with ketamine (K) and xylazine (X) in 0.9% NaCl (100 and 10 mg/kg BW, respectively) according to the anesthesia and euthanasia guidelines for laboratory rodents. The unconscious (a painless state) rats were then laparotomized, and their blood was collected from the caudal vena cava into heparinized tubes to obtain blood plasma via centrifugation (350× *g*, 10 min, 4 °C). Plasma samples were kept frozen at −70 °C until analysis. After blood collection, the rats were euthanized by cervical dislocation to confirm death. The selected internal organs and tissues (epididymal fat pad, liver, small intestine, cecum) were removed, weighed, frozen in liquid nitrogen (−196 °C), and stored in low-temperature freezers at −70 °C.

As soon as possible after termination (10–15 min) and laparotomy, samples of fresh ileal, cecal, and colonic digesta were analyzed to determine the pH (ileal, cecal, and colonic), concentrations of cecal ammonia and cecal short-chain fatty acids (SCFAs). The remaining digesta samples were transferred to microfuge tubes and stored at −70 °C until analyses of bacterial enzymatic activity. The pH values in the digesta were measured with the aid of a pH/ion meter (model 301, Hanna Instruments, Vila do Conde, Portugal). Cecal ammonia was captured in a H_3_BO_3_ solution in Conway dishes and analyzed by titration with sulfuric acid according to a previously reported protocol [9]. The cecal concentrations of SCFAs were analyzed by gas chromatograph (GC) (Shimadzu GC-2010, Kyoto, Japan) as reported in detail by Fotschki et al. (2014) [25]. Briefly, the samples were mixed with the internal standard formic acid, diluted with deionized water and centrifuged at 7211× *g* for 10 min. The following equipment and conditions were utilized: capillary column (SGE BP21, 30 m × 0.53 mm); oven temperature, initially 85 °C, increasing at a rate of 8 °C per minute to reach 180 °C, where it was held for 3 min; flame ionization detector temperature, 180 °C; injector temperature, 85 °C; and sample volume, 1 μL. Pure acetic, propionic, butyric, isobutyric, isovaleric and valeric acids were obtained from Sigma Co. (Poznan, Poland), and their mixture was used to create a standard curve for calculation of the amount of each individual acid. This additional set of pure acids was included in each GC run after every five samples to maintain calibration. The putrefactive short-chain fatty acids (PSCFAs) were calculated as the sum of isobutyric, isovaleric and valeric acids. The analyses were performed in duplicate.

In the collected blood plasma, triglycerides (TGs), total cholesterol (TC), the fractions of HDL cholesterol (HDL) and LDL cholesterol (LDL) and the activities of AST, ALP and ALT were estimated using a biochemical analyzer (Pentra C200, Horiba, Tokyo, Japan). The concentrations of IL-6, IL-10 and TNFα were determined using commercial ELISA kits (Sigma Aldrich, USA). The atherogenic index of plasma (AIP) was calculated using the formula lg(TG/HDL). The atherogenic coefficient (AC) was calculated as (TC-HDL)/HDL. Additionally, the TG/HDL, LDL/HDL, and TC/HDL ratios were determined. After storing the livers at −70 °C, hepatic lipids were extracted according to the method described by Folch et al. (1957) [26]. Following extraction, TC and TGs were determined enzymatically using commercial kits (Alpha Diagnostics Ltd., Warsaw, Poland). Total RNA was extracted from the liver samples using TRI Reagent (Sigma-Aldrich, Saint Louis, MO, USA). The quantity and quality of RNA was examined via a NanoDrop 1000 instrument (Thermo Scientific, Waltham, MA, USA). cDNA was synthesized from 500 ng of total RNA using a High-Capacity cDNA Reverse Transcription Kit with RNase Inhibitor (Applied Biosystem, Waltham, MA, USA). To measure the hepatic mRNA expression of peroxisome proliferator-activated receptors alpha (PPARα) and gamma (PPARγ), and SREBP-1c, single tube TaqMan^®^ Gene Expression Assays (Life Technologies, Santa Clara, CA, USA) were used. Amplification was performed using a 7900HT Fast Real-Time PCR System under the following conditions: initial denaturation for 10 min at 95 °C, followed by 40 cycles of 15 s at 95 °C and 1 min at 60 °C. Each run included a standard curve based on aliquots of pooled liver RNA. All samples were analyzed in duplicate. The mRNA expression levels were normalized to that of the reference gene β-actin (ACTB). The analyses of polyphenolic metabolites in plasma were carried out according to the modified method described by Płatosz et al. (2022) [27]. Plasma samples were vortexed, sonicated, and placed on SPE cartridges (StrataTM-X, Phenomenex, Torrance, CA, USA) conditioned with methanol/trifluoroacetic acid (MeOH/TFA) (99.6/0.4; *v*/*v*). Then, the cartridges were flushed with a 0.4% TFA aqueous solution, and the compounds were eluted with MeOH/TFA (99.6/0.4; *v*/*v*). The obtained supernatants were evaporated to dryness under a nitrogen stream at 35 °C, dissolved in H2O/MeOH/formic acid (FA) (94.1/5/0.9; *v*/*v*), vortexed, sonicated, filtered through Micro-Spin PES Filter Tubes (Ciro Manufacturing, Deerfield Beach, USA), and centrifuged (20 min, 13,200× *g*, 4 °C, 5415R, Eppendorf, Hamburg, Germany). The solutions were subsequently injected into a micro-HPLC system (LC-200, Eksigent, Vaughan, Canada) coupled to a triple quadrupole mass spectrometer (QTRAP 5500, AB Sciex, Vaughan, Canada) with an ion trap and electrospray ionization (ESI) source. Chromatographic analysis was conducted with a HALO C18 column (0.5 mm × 100 mm × 2.7 µm, Eksigent, Vaughan, Canada) at 45 °C and a flow rate of 15 µL/min. Elution was carried out using a gradient solvent system including solvent A (0.9% (*v*/*v*) formic acid aqueous solution) and solvent B (0.9% (*v*/*v*) formic acid acetonitrile solution). The gradient used was as follows: 0.4% B (0–0.5 min), 0.4–95% B (0.5–2.0 min), 95% B (2.0–3.0 min), 95–0.5% B (3.0–3.5 min) and 0.4% B (3.5–4.0 min). Optimal identification of the analyzed compounds was achieved under the following ESI-MS/MS conditions: positive ionization; curtain gas, 25 L/min; collision gas; ion spray voltage, 5300 V; temperature, 350 °C; ion source gas 1, 35 L/min; ion source gas 2, 30 L/min; declustering potential, 70–100 V; entrance potential, 10 V; collision energy, 30–40 eV; and collision cell exit potential, 15–20 V. Identification was based on comparison of the retention time of the analyte and the presence of the respective parent and daughter ion pairs (multiple reaction monitoring mode) with data obtained after analysis of authentic standards and/or published data. Quantitative analysis was carried out with external standards (0.01–1 μM) that had been used to construct linear calibration curves with correlation coefficients of 0.994–0.997. All analyses were performed in triplicate for each sample.

### 2.5. Statistical Analysis

In the tables and figures, the results are presented as the means (*n* = 8) with standard deviations (SDs). One-way ANOVA and a post hoc Duncan’s test were applied to assess significant differences among all four experimental groups. One-way ANOVA required the normal distribution of samples, so the data were checked for normality via the Shapiro–Wilk test. The blood plasma concentrations of the polyphenolic metabolites were analyzed in the HF + 0.1PP and HF + 0.3PP groups, so a t test was applied to determine the statistical significance of the difference at *p* < 0.05. STATISTICA ver. 12.0 software (StatSoft Corp., Krakow, Poland) was used for analysis.

## 3. Results

The rats in all three dietary high-fat treatment groups (HF, HF + 0.1PP, HF + 0.3PP) had significantly increased daily body weight gains compared to the rats in the control group (Table 3; *p* < 0.05).

The daily diet intake was the highest in HF + 0.1PP rats (*p* < 0.05 vs. the HF group). The calculated dietary intake needed per gram of BW gain was significantly higher in the control (C) group than in the three HF groups (*p* < 0.05). The NMR analyses showed that the HF, HF + 0.1PP and HF + 0.3PP groups of rats surpassed the C rats with regard to fat tissue percentage (*p* < 0.05). The highest lean tissue percentage followed dietary treatment C (*p* < 0.05 vs. all remaining groups). The HF and HF + 0.1PP treatments resulted in the greatest relative weight (calculated per 100 g BW) of epididymal white adipose tissue (eWAT; *p* < 0.05 vs. C). The eWAT weight in the HF + 0.3PP animals did not differ significantly in comparison to the control and to the remaining HF groups (*p* > 0.05). The relative weight of the small intestinal tissue and small intestinal digesta pH did not differ significantly among treatments (*p* > 0.05).

The relative cecal tissue weight tended to be lower in the HF and HF + 0.1PP rats than in the C and HF + 0.3PP animals (*p* = 0.052; Table 4).

The highest cecal ammonia concentration followed dietary HF treatment (*p* < 0.05 vs. C rats), while dietary supplementation with both extract doses caused a drop in cecal ammonia levels (*p* > 0.05 vs. C, HF). The cecal digesta pH value was similar in the C and HF groups (values 7.09–7.11), while the HF + 0.3PP treatment caused a significant decrease in cecal digesta pH (6.79; *p* < 0.05 vs. C, HF). Significant changes in microbial activity related to the production of SCFAs were also observed. A significant decrease in the cecal acetic acid concentration followed dietary treatment with the HF and HF + 0.1PP diets (*p* < 0.05 vs. C and HF + 0.3PP groups). The lowest concentration of propionic acid in the cecal digesta was observed in the HF rats (*p* < 0.05 vs. C and HF + 0.1PP), while the highest C3 concentration was noted in the control group (*p* < 0.05 vs. all other groups). The cecal concentrations of isobutyric, butyric, and isovaleric acids were significantly decreased in all three high-fat treatment groups (HF, HF + 0.1PP, HF + 0.3PP) compared to the control (C) animals (*p* < 0.05). The lowest cecal valeric acid concentration was found in the HF and HF + 0.3PP dietary treatment groups (*p* < 0.05 vs. C and HF + 0.1PP), and the highest C5 concentration was in the C group (*p* < 0.05 vs. all remaining groups). The cecal concentration of PSCFAs (the sum of isobutyric, isovaleric and valeric acids) was the lowest in the cecal digesta of the HF + 0.3PP rats (*p* < 0.05 vs. C and HF + 0.1PP). The highest PSCFA concentration in the cecal digesta was noted in the control (C) animals (*p* < 0.05 vs. all other groups). As a result of all the above mentioned changes, the total SCFA concentration in the cecum was significantly lowered by all three HF treatments in comparison to the C group (*p* < 0.05). The highest and lowest colonic digesta pH values were noted in the HF and HF + 0.1PP groups, respectively (*p* < 0.05 vs. HF + 0.1PP and *p* < 0.05 vs. HF, respectively).

The relative liver weights were significantly elevated in the HF diet group and both groups fed HF diets supplemented with raspberry extract (*p* < 0.05 vs. C; Table 5).

The hepatic fat content as well as TC and TG contents in the liver tissue were substantially elevated in all three HF groups of rats compared to the C animals (*p* < 0.05). Additionally, the hepatic TG content in the HF group was significantly higher than that in the HF + 0.3PP group (*p* < 0.05). The thiobarbituric acid reactive substances (TBARS) content in the liver of the HF rats was significantly higher than that in all other groups (*p* < 0.05 vs. C, HF + 0.1PP, HF + 0.3PP). The TC and LDL concentrations in blood plasma did not differ significantly among groups (*p* > 0.05). The HF rats had significantly lower plasma HDL concentrations than the other groups (*p* < 0.05 vs. C and both groups supplemented with raspberry extract). The plasma TG concentration did not differ statistically among treatments (*p* > 0.05), but the TG/HDL ratio tended (*p* = 0.054) to be increased in HF rats. The LDL/HDL and TC/HDL ratios were significantly elevated in the HF group (*p* < 0.05 vs. C, HF + 0.1PP and *p* < 0.05 vs. C, HF + 0.1PP, HF + 0.3PP groups, respectively). The AC value, calculated as (TC-HDL)/HDL, was the highest in the HF animals (*p* < 0.05 vs. the remaining groups). The AIP value (lg(TG/HDL)) was enhanced by HF dietary treatment compared to the C treatment (*p* < 0.05). The activities of aspartate transaminase (AST), alanine transaminase (ALT), and alkaline phosphatase (ALP) in the blood plasma were considerably (*p* < 0.05) enhanced in all three HF treatment groups (HF and both groups supplemented with raspberry extract) in comparison to the control rats fed a low-fat diet.

**Figure 1:**
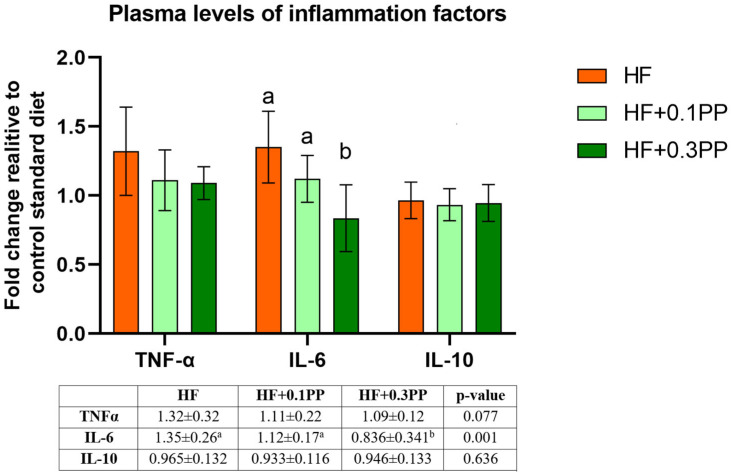
The levels of TNFα, IL-6, and IL-10 in the plasma of rats fed experimental diets (expressed as fold change relative to the control). The values are the means ± SEMs. HF, group fed a high-fat diet; HF + 0.1PP, group fed a high-fat diet supplemented with 0.1% raspberry polyphenolic extract; HF + 0.3PP, group fed a high-fat diet supplemented with 0.3% raspberry polyphenolic extract. Mean values with different superscript letters (a or b) are different at *p* < 0.05 (post hoc test). TNFα, tumor necrosis factor α; IL-6, interleukin 6; IL-10, interleukin 10.

The concentration of tumor necrosis factor alpha (TNFα) in blood plasma tended to be lower in the HF + 0.3PP group (*p* = 0.077; Figure 1). The IL-6 level significantly decreased in the HF + 0.3PP rats in comparison to the HF and HF + 0.1PP animals (*p* < 0.05; Figure 1). 

**Figure 2:**
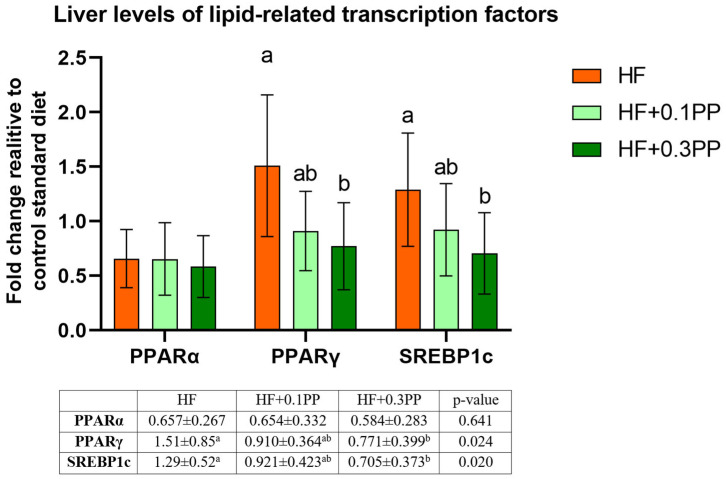
mRNA expression of PPARα, PPARγ, and SREBP-1c in the livers of rats fed experimental diets (expressed as fold change relative to the control). The values are the means ± SEMs. HF, group fed a high-fat diet; HF + 0.1PP, group fed a high-fat diet supplemented with 0.1% raspberry polyphenolic extract; HF + 0.3PP, group fed a high-fat diet supplemented with 0.3% raspberry polyphenolic extract. Mean values with different superscript letters (a or b) are different at *p* < 0.05 (post hoc test). PPARα, peroxisome proliferator-activated receptor alpha; PPARγ, peroxisome proliferator-activated receptor gamma; SREBP-1c, sterol regulatory element-binding protein 1c.

The PPARγ and SREBP-1c mRNA expression levels in the liver tissue (expressed as fold change in relation to those in the control group) were significantly decreased after HF + 0.3PP treatment compared to those in the HF animals (*p* < 0.05 vs. HF; Figure 2).

**Figure 3:**
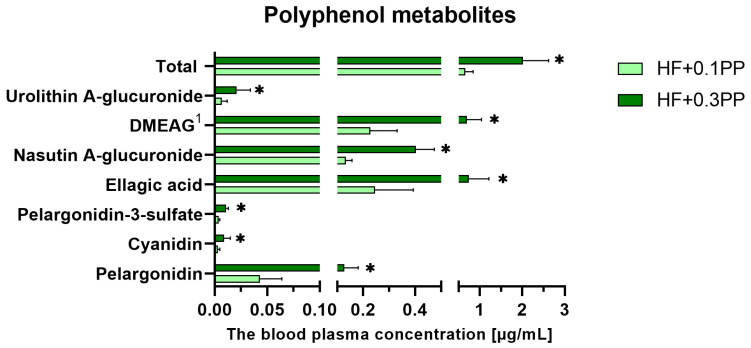
Blood plasma concentrations of polyphenolic metabolites in rats fed diets supplemented with raspberry extract. The values are the means ± SEMs. HF + 0.1PP, group fed a high-fat diet supplemented with 0.1% raspberry polyphenolic extract; HF + 0.3PP, group fed a high-fat diet supplemented with 0.3% raspberry polyphenolic extract. * Mean values are significantly different (*p* < 0.05; *t* test). ^1^ ellagic acid dimethyl ether glucuronide.

The two groups of rats fed diets supplemented with the raspberry extract were compared by t test regarding blood plasma polyphenolic metabolite concentrations (Figure 3).

Considering all detected metabolites, the contents were significantly higher in the HF + 0.3PP group than in the HF + 0.1PP group (i.e., pelargonidin, cyaniding, pelargonidin-3-sulfate, ellagic acid, nasutin A-glucuronide, ellagic acid dimethyl ether glucuronide (DMEAG), and urolithin A-glucuronide), as were the total content of all metabolites (*p* < 0.05).

## 4. Discussion

One of the most popular berries worldwide is raspberries. These fruits are mostly consumed fresh, or they are processed into jams and confitures and used as ingredients in various foods. In addition to their nutritional value, raspberries are also a great source of phenolic compounds with well-known health-promoting properties, e.g., ETs, ACYs and flavanols [1]. In this study, the polyphenolic extract obtained from raspberry pomace contained mostly ETs (81%), flavanols in smaller quantities (18%) and ACYs (1%). Numerous studies have shown that diets enriched with raspberry polyphenols are beneficial to prevent obesity, inflammation and other metabolic disorders [13,21,28]. Recent studies on polyphenols have also focused on their regulation of intestinal microbial activity and profile to prevent or regulate high-fat diet-induced dysbiosis in the gastrointestinal tract [19,29]. Microbiological in vitro studies have shown that supplementation with ETs may exert a prebiotic effect by stimulating the growth of *Bifidobacteria* and *Lactobacilli* and inhibiting the groups *Enterobacteriaceae*, *Clostridia*, and *Bacteroides fragilis* in a dose-dependent manner [30]. Indeed, in our study, the strongest effect high-fat diet-related microbial disorder regulation was observed when the diet was supplemented with the higher concentration of polyphenols from raspberries. This effect was associated with a downwards trend in the concentration of ammonia and a considerably lower level of PSCFAs in the cecum. Changes in the cecal levels of ammonia and PSCFAs might be associated with decreased bacterial breakdown of urea, undigested protein substances and higher dietary protein utilization. The observed changes in microbial activity might be considered beneficial because an increased concentration of ammonia in the lower bowel can induce disturbance in the functioning of intestinal cells, e.g., alterations to nucleic acid synthesis and a decrease in resistance to viral infections [31]. The higher raspberry polyphenol supplementation dose also favorably lowered the pH value in the cecum. A lower pH of the intestine digesta might inhibit the growth of pathogens that colonize neutral or slightly alkaline environments and promote the growth of commensal bacterial species, e.g., *Lactobacillus* and *Bifidobacterium*, which are able to elevate the concentration of SCFAs in the gastrointestinal tract [32]. In this study, the total concentration of SCFAs in the cecum was not changed after the addition of raspberry polyphenols to the high-fat diet. However, in terms of the SCFA profile, considerably more acetic acid was produced in the group given the higher dose of polyphenolic extract. This effect might be associated with the presence of more raspberry polyphenols and their metabolites, which may affect the growth and activity of gastrointestinal microbiota and thus modulate SCFA production [33].

Changes at the intestinal level also affect the liver and systemic parameters. Yamashita et al. (2016) [34] performed nutritional studies on rats and showed that supplementation with acetic acid reduced liver lipid accumulation and abdominal fat and thus activated mechanisms that work against the development of obesity. In our study, supplementation with polyphenols from raspberries had similar effects on the livers of rats fed a high-fat diet. Among all groups of rats fed high-fat diets, the lowest average liver fat accumulation and total cholesterol were observed after treatment with the higher dose of polyphenols; however, these data were not considerably different. The higher dose of polyphenols considerably reduced the eWAT weight and TG levels in the liver and improved hepatic antioxidative potential by decreasing the level of TBARS. The effect on lipid metabolism and oxidative parameters in the liver was probably associated with increased microbial production of polyphenolic metabolites in the intestine, and thus, elevation of these metabolites in the blood plasma was observed in this study. Recent studies suggest that ellagic acid, ACYs and their derivatives might regulate the molecular mechanisms of the liver to work against high-fat diet-induced obesity by diminishing the expression of the hepatic molecular factors SREBP-1c and PPARγ, which are involved in the storage of fat and TGs [35,36,37]. Wu et al. (2021) [18] also showed that raspberry polyphenolic extracts can favorably attenuate ROS accumulation in hepatic stellate cells and regulate lipid metabolism and hepatic fibrosis through the Nrf2 and PPARγ pathways. In our study, regulation of SREBP-1c and PPARγ expression in the liver was observed only when the high-fat diet was supplemented with the higher dose of polyphenolic extract. The favorable effect of increased supplementation was also reflected in the reduction in the proinflammatory factor IL-6 in the plasma. Umesalma and Sudhandiran (2010) [38], in nutritional experiments on rats with induced colon cancer, also observed that administration of ellagic acid downregulates IL-6 and that the effect is related to inhibition of NF-kappaB. Additionally, a nutritional study with ACYs in participants with dyslipidemia showed that supplementation with more of these polyphenols exerts a stronger effect on the reductions in IL-6 and TBARS in the serum [7]. Another study on death-induced obesity-related disorders found that enrichment with polyphenols from raspberries might also improve the blood lipid profile by elevating HDL cholesterol and reducing LDL cholesterol and TGs [13]. Our study showed that the main effect that the polyphenols from raspberry pomace (regardless of the dose) had on blood lipid profile regulation was focused on the elevation in HDL cholesterol and thus a favorable reduction in the atherogenic index and TC/HDL ratio.

## 5. Conclusions

In conclusion, both dietary doses of the raspberry extract obtained from pomace applied for 30 days to rats fed high-fat diets caused beneficial health changes in terms of lipid metabolism in the liver and blood plasma, manifested by, among other factors, the decreased TBARS content in the liver, enhanced plasma HDL concentration, and reduced plasma TC/HDL ratio and AC value to the levels observed in the rats fed a standard diet. With respect to the accepted hypothesis, the group of rats fed a high-fat diet with the higher (0.3% of the diet) raspberry polyphenol dose received more benefits than the rats fed the lower dose (0.1% of the diet) in terms of eWAT weight, cecal acetic acid concentration, plasma IL-6, hepatic TGs, PPARγ, and SREBP-1c. These changes might be partly ascribed to the enhanced blood plasma concentration of ET and ACY bioactive metabolites, i.e., pelargonidin, cyaniding, pelargonidin-3-sulfate, ellagic acid, nasutin A-glucuronide, ellagic acid dimethyl ether glucuronide and urolithin A-glucuronide. In summary, the use of raspberry polyphenol extract from pomace should be considered a valuable, affordable, and suitable way to enrich our diet with an effective amount of bioactive molecules. Furthermore, this experiment was performed in model animals; therefore, the use of raspberry polyphenolic extracts as a functional additive to food should also be verified by human studies.

## Figures and Tables

**Table 1:** Chemical composition of the raspberry polyphenol extract.

Compound	
Basic components (*n* = 3), g/100 g	
Dry matter (AOAC 940.26)	94.79 ± 0.18
Protein (AOAC 920.152)	4.74 ± 0.45
Fat (AOAC 930.09)	0.51 ± 0.04
Ash	2.11 ± 0.06
TDF (AOAC 985.29)	0.00 ± 0.00
Total polyphenols	47.80 ± 1.06
Saccharides	
Saccharose	1.60 ± 0.00
Glucose	8.90 ± 0.60
Fructose	9.90 ± 0.60
Polyphenols (*n* = 3), mg/100 g	
Total polyphenols (TPH)	47804.8 ± 1060.5
Ellagitannins (ETs)	
Sanguiin H-6	16975.8 ± 350.4
Sanguiin H-6 minus a gallic acid moiety^a^	221.6 ± 9.7
Sanguiin H-6 plus a gallic acid moiety^a^	356.3 ± 22.4
Sanguiin H-10 isomer 1 ^a^	466.4 ± 14.0
Sanguiin H-10 isomer 2 ^a^	533.0 ± 20.7
Sanguiin H-10 isomer 3 ^a^	301.2 ± 10.7
Lambertianin C	18314.0 ± 1172.6
Lambertianin C minus ellagic acid moiety 1 ^b^	764.1 ± 28.8
Lambertianin C minus ellagic acid moiety 2 ^b^	196.4 ± 10.9
Lambertianin C minus ellagic acid moiety 3 ^b^	426.6 ± 6.7
Ellagic acid pentose conjugate ^c^	171.7 ± 11.4
Ellagic acid (EA)	196.7 ± 18.1
Total ETs	38088.9 ± 1503.6
Total EACs	368.3 ± 29.4
Total ETs + EACs	38923.6 ± 1547.0
Flavanols	
Total flavanols	8371.7 ± 486.5
(+)-Catechin	208.0 ± 5.4
(-)-Epicatechin	343.5 ± 5.5
Proanthocyanidins	7820.2 ± 475.7
Anthocyanins (ACYs)	
Cyanidin-3-*O*-spohoroside ^d^	314.6 ± 11.5
Cyanidin-3-*O*-glucosyl-rutinoside ^d^	27.0 ± 0.7
Cyanidin-3-*O*-glucoside	152.0 ± 0.9
Cyanidin-3-*O*-rutinoside ^d^	11.5 ± 0.1
Pelargonidin-3-*O*-glucoside ^d^	4.5 ± 0.3
Total ACYs	509.6 ± 10.9

Values are expressed as the mean ± standard deviation (mg/100 g); *n*, number of measurements; mDP, mean degree of polymerization; Total EACs, the total contents of ellagic acid and its conjugates. ^a^ The contents of these substances were calculated based on the sanguiin H-6 standard. ^b^ The contents of these substances were calculated based on the lambertianin C standard. ^c^ The contents of these substances were calculated based on ellagic acid standards. ^d^ The contents of anthocyanins were calculated based on the cyanidin-3-O-glucoside standard.

**Table 2:** Compositions of the diets used in the in vivo rat experiment, g/100 g.

	C	HF	HF + 0.1PP	HF + 0.3PP
Casein ^1^	20.0	20.0	20.0	20.0
DL-Methionine	0.30	0.30	0.30	0.30
Cellulose ^2^	5.0	3.0	3.0	3.0
Choline chloride	0.20	0.20	0.20	0.20
Cholesterol	0.30	1.0	1.0	1.0
Vitamin mix ^3^	1.0	1.0	1.0	1.0
Mineral mix ^4^	3.5	3.5	3.5	3.5
Rapeseed oil	2.0	2.0	2.0	2.0
Lard	6.0	23.0	23.0	23.0
Raspberry polyphenolic extract	0.00	0.00	0.21	0.64
Saccharose	10.0	10.0	10.0	10.0
Maize starch ^5^	51.7	36.0	35.79	35.36
kcal%, calculated				
Protein	19.6	15.4	15.4	15.4
Carbohydrate	59.5	35.2	35.2	35.2
Fat	20.9	49.4	49.4	49.4

^1^ Casein preparation: crude protein 89.7%, crude fat 0.3%, total dietary fiber 0%, ash 2.0%, and water 8.0%. ^2^ α-Cellulose (SIGMA, Poznan, Poland) was the main source of dietary fiber. ^3^ AIN-93G-VM (Reeves, 1997, [24]), g/kg mix: 3.0 nicotinic acid, 1.6 Ca pantothenate, 0.7 pyridoxine HCl, 0.6 thiamine HCl, 0.6 riboflavin, 0.2 folic acid, 0.02 biotin, 2.5 vitamin B-12 (cyanocobalamin, 0.1% in mannitol), 15.0 vitamin E (all-rac-α-tocopheryl acetate, 500 IU/g), 0.8 vitamin A (all-trans-retinyl palmitate, 500,000 IU/g), 0.25 vitamin D-3 (cholecalciferol, 400,000 IU/g), 0.075 vitamin K-1 (phylloquinone), 974.655 powdered sucrose. ^4^ Mineral mix, g/kg mix: 357 calcium carbonate anhydrous (CaCO3), 196 dipotassium phosphate (K2HPO4), 70.78 potassium citrate (C6H5K3O7), 74 sodium chloride (NaCl), 46.6 potassium sulfate (K2SO4), 24 magnesium oxide (MgO), 18 microelement mixture, starch to 1 kg = 213.62. Microelement mixture, g/kg mix: 31 iron (III) citrate (16.7% Fe), 4.5 zinc carbonate (ZnCO3) (56% Zn), 23.4 manganese (II) carbonate (MnCO3) (44.4% Mn), copper carbonate (CuCO3) (55.5% Cu), 0.04 potassium iodide (KI), citric acid (C6H8O7) to 100 g. ^5^ Maize starch preparation: crude protein 0.6%, crude fat 0.9%, ash 0.2%, total dietary fiber 0%, and water 8.8%.

**Table 3:** Growth parameters and dietary intake of the rats fed experimental diets and nuclear magnetic resonance (NMR) analysis of selected tissues.

	C	HF	HF + 0.1PP	HF + 0.3PP	*p* Value
Initial BW, g	376 ± 19.4	385 ± 29.5	388 ± 32.3	380 ± 31.2	0.458
BW gain, g/day	1.70 ± 0.52 ^b^	2.97 ± 0.43 ^a^	3.42 ± 0.63 ^a^	3.14 ± 0.45 ^a^	<0.001
Daily diet intake, g	17.4 ± 1.47 ^ab^	16.5 ± 1.27 ^b^	18.0 ± 1.32 ^a^	17.0 ± 1.19 ^ab^	0.042
Intake per 1 g gain, g	10.9 ± 2.68 ^a^	5.63 ± 0.72 ^b^	5.37 ± 0.74 ^b^	5.50 ± 0.61 ^b^	<0.001
Fat tissue ^1^, %	19.8 ± 3.08 ^b^	29.8 ± 1.45 ^a^	30.4 ± 3.10 ^a^	28.7 ± 2.97 ^a^	<0.001
Lean tissue ^1^, %	61.4 ± 2.68 ^a^	54.3 ± 1.08 ^b^	54.1 ± 2.97 ^b^	54.0 ± 3.06 ^b^	<0.001
eWAT ^2^, g/100 g BW	3.66 ± 1.47 ^b^	5.09 ± 0.94 ^a^	4.75 ± 0.60 ^a^	4.53 ± 0.48 ^ab^	0.009
Small intestine					
Tissue, g/100 g BW	0.561 ± 0.084	0.557 ± 0.072	0.583 ± 0.065	0.625 ± 0.067	0.105
pH of contents	6.96 ± 0.24	7.00 ± 0.23	6.85 ± 0.33	6.83 ± 0.23	0.240

The results are presented as the mean ± SD (standard deviation). C, group fed the control low-fat diet; HF, group fed the high-fat diet; HF + 0.1PP, group fed the high-fat diet supplemented with 0.1% raspberry polyphenolic extract; HF + 0.3PP, group fed the high-fat diet supplemented with 0.3% raspberry polyphenolic extract. BW, body weight; ^1^ analysis by nuclear magnetic resonance (NMR); ^2^ eWAT, epididymal white adipose tissue. Mean values with different superscript letters within a row (a or b) are different at *p* < 0.05 (post hoc test).

**Table 4:** Large bowel parameters of and microbial SCFA production in rats fed experimental diets.

	C	HF	HF + 0.1PP	HF + 0.3PP	*p* Value
Cecum					
Tissue, g/100 g BW	0.164 ± 0.035	0.134 ± 0.018	0.132 ± 0.027	0.161 ± 0.031	0.052
Ammonia, mg/g	0.232 ± 0.028 ^b^	0.319 ± 0.067 ^a^	0.300 ± 0.069 ^ab^	0.286 ± 0.073 ^ab^	0.016
pH of digesta	7.11 ± 0.23 ^a^	7.09 ± 0.22 ^a^	6.97 ± 0.22 ^ab^	6.79 ± 0.25 ^b^	0.021
SCFAs, µmol/g					
Acetic acid (C_2_)	33.5 ± 3.38 ^a^	26.4 ± 5.24 ^b^	25.8 ± 4.61 ^b^	30.9 ± 2.77 ^a^	0.001
Propionic acid (C_3_)	10.2 ± 0.87 ^a^	7.84 ± 0.97 ^c^	9.02 ± 0.85 ^b^	8.53 ± 1.15 ^bc^	<0.001
Isobutyric acid(C_4i_)	0.99 ± 0.31 ^a^	0.63 ± 0.13 ^b^	0.65 ± 0.09 ^b^	0.60 ± 0.21 ^b^	0.001
Butyric acid (C_4_)	4.13 ± 1.03 ^a^	1.86 ± 0.56 ^b^	1.78 ± 0.81 ^b^	1.80 ± 0.68 ^b^	<0.001
Isovaleric acid (C_5i_)	1.37 ± 0.19 ^a^	0.78 ± 0.14 ^b^	0.97 ± 0.22 ^b^	0.79 ± 0.18 ^b^	<0.001
Valeric acid (C_5_)	1.06 ± 0.08 ^a^	0.62 ± 0.09 ^c^	0.80 ± 0.15 ^b^	0.59 ± 0.14 ^c^	<0.001
PSCFAs	3.42 ± 0.35 ^a^	2.03 ± 0.27 ^bc^	2.41 ± 0.41 ^b^	1.98 ± 0.44 ^c^	<0.001
Total SCFAs	51.2 ± 4.16 ^a^	38.1 ± 6.33 ^b^	39.0 ± 6.15 ^b^	43.2 ± 3.65 ^b^	<0.001
**Colon**					
pH of the digesta	7.04 ± 0.27 ^ab^	7.20 ± 0.20 ^a^	6.84 ± 0.28 ^b^	6.92 ± 0.31 ^ab^	0.021

The results are presented as the mean ± SD (standard deviation). C, group fed the control low-fat diet; HF, group fed the high-fat diet; HF + 0.1PP, group fed the high-fat diet supplemented with 0.1% raspberry polyphenolic extract; HF + 0.3PP, group fed the high-fat diet supplemented with 0.3% raspberry polyphenolic extract. Mean values with different superscript letters within a row (a, b or c) are different at *p* < 0.05 (post hoc test).

**Table 5:** Liver and blood plasma biochemical parameters of rats fed experimental diets.

	C	HF	HF + 0.1PP	HF + 0.3PP	*p* Value
Liver					
Weight	2.93 ± 0.14 ^b^	3.40 ± 0.23 ^a^	3.48 ± 0.26 ^a^	3.48 ± 0.43 ^a^	<0.001
Hepatic fat, %	18.8 ± 3.06 ^b^	34.4 ± 4.16 ^a^	34.1 ± 5.70 ^a^	29.9 ± 4.98 ^a^	<0.001
TC, mg/g	8.11 ± 1.51 ^b^	10.3 ± 1.00 ^a^	9.89 ± 1.18 ^a^	9.10 ± 0.76 ^a^	0.001
TGs, mg/g	12.5 ± 1.42 ^c^	18.8 ± 2.05 ^a^	17.9 ± 3.80 ^ab^	15.6 ± 3.01 ^b^	<0.001
TBARS, ng/g	587 ± 47.7 ^b^	768 ± 96.9 ^a^	662 ± 80.4 ^b^	631 ± 70.7 ^b^	<0.001
**Blood plasma**					
TC, mmol/L	2.52 ± 0.51	2.87 ± 0.58	2.59 ± 0.41	2.70 ± 0.58	0.232
HDL, mmol/L	0.495 ± 0.066 ^a^	0.404 ± 0.069^b^	0.488 ± 0.054 ^a^	0.469 ± 0.028 ^a^	0.005
LDL, mmol/L	0.329 ± 0.069	0.498 ± 0.236	0.394 ± 0.151	0.508 ± 0.242	0.091
TGs, mmol/L	0.756 ± 0.132	0.824 ± 0.155	0.850 ± 0.193	0.841 ± 0.230	0.354
TG/HDL ratio	1.55 ± 0.30	2.17 ± 0.92	1.75 ± 0.42	1.79 ± 0.44	0.054
LDL/HDL ratio	0.66 ± 0.08 ^b^	1.31 ± 0.73 ^a^	0.81 ± 0.29 ^b^	1.08 ± 0.52 ^ab^	0.015
TC/HDL ratio	5.07 ± 0.54 ^b^	7.48 ± 2.83 ^a^	5.32 ± 0.68 ^b^	5.74 ± 1.12 ^b^	0.008
AC	4.07 ± 0.54 ^b^	6.48 ± 2.83 ^a^	4.32 ± 0.68 ^b^	4.74 ± 1.12 ^b^	0.008
AIP	0.182 ± 0.083 ^b^	0.310 ± 0.149 ^a^	0.234 ± 0.097 ^ab^	0.242 ± 0.097 ^ab^	0.039
AST, U/L	69.5 ± 6.90 ^b^	167 ± 26.9 ^a^	167 ± 52.5 ^a^	142 ± 22.5 ^a^	<0.001
ALP, U/L	41.1 ± 35.3 ^b^	97.2 ± 21.9 ^a^	103 ± 47.0 ^a^	96.7 ± 35.3 ^a^	0.003
ALP, U/L	56.8 ± 8.95 ^b^	96.5 ± 22.8 ^a^	89.4 ± 13.2 ^a^	85.6 ± 29.8 ^a^	0.001

C, group fed the control low-fat diet; HF, group fed the high-fat diet; HF + 0.1PP, group fed the high-fat diet supplemented with 0.1% raspberry polyphenolic extract; HF + 0.3PP, group fed the high-fat diet supplemented with 0.3% raspberry polyphenolic extract (1 g/100 g BW). Mean values with different superscript letters within a row (a, b or c) are different at *p* < 0.05 (post hoc test). AC, atherogenic coefficient (TC-HDL)/HDL); AIP, atherogenic index of plasma (lg(TG/HDL)); TC, total cholesterol; TGs, triglycerides; AST, aspartate transaminase; ALT, alanine transaminase; ALP, alkaline phosphatase; TBARS, thiobarbituric acid reactive substances.

## Data Availability

Not applicable.
